# *In vitro* and *in vivo* anti-tumor effect of metformin as a novel therapeutic agent in human oral squamous cell carcinoma

**DOI:** 10.1186/1471-2407-12-517

**Published:** 2012-11-14

**Authors:** Qingqiong Luo, Dan Hu, Shuiqing Hu, Ming Yan, Zujun Sun, Fuxiang Chen

**Affiliations:** 1 Department of Clinical Laboratories, Ninth People’s Hospital Affiliated to Shanghai Jiao Tong University School of Medicine, 639 Zhizaoju Road, Shanghai, 200011, China; 2 Department of Oral and Maxillofacial Surgery, Ninth People’s Hospital, Shanghai Jiao Tong Universtity School of Medicine, Shanghai, China

**Keywords:** Metformin, Oral squamous cell carcinomas, Cell cycle, Cyclin D1, Apoptosis

## Abstract

**Background:**

Metformin, which is widely used as an antidiabetic agent, has recently been reported to reduce cancer risk and improve prognosis in certain malignancies. However, the specific mechanisms underlying the effect of metformin on the development and progression of several cancers including oral squamous cell carcinoma (OSCC) remain unclear. In the present study, we investigated the effects of metformin on OSCC cells *in vitro* and *in vivo*.

**Methods:**

OSCC cells treated with or without metformin were counted using a hemocytometer. The clonogenic ability of OSCC cells after metformin treatment was determined by colony formation assay. Cell cycle progression and apoptosis were assessed by flow cytometry, and the activation of related signaling pathways was examined by immunoblotting. The *in vivo* anti-tumor effect of metformin was examined using a xenograft mouse model. Immunohistochemistry and TUNEL staining were used to determine the expression of cyclin D1 and the presence of apoptotic cells in tumors from mice treated with or without metformin.

**Results:**

Metformin inhibited proliferation in the OSCC cell lines CAL27, WSU-HN6 and SCC25 in a time- and dose-dependent manner, and significantly reduced the colony formation of OSCC cells *in vitro.* Metformin induced an apparent cell cycle arrest at the G0/G1 phase, which was accompanied by an obvious activation of the AMP kinase pathway and a strongly decreased activation of mammalian target of rapamycin and S6 kinase. Metformin treatment led to a remarkable decrease of cyclin D1, cyclin-dependent kinase (CDK) 4 and CDK6 protein levels and phosphorylation of retinoblastoma protein, but did not affect p21 or p27 protein expression in OSCC cells. In addition, metformin induced apoptosis in OSCC cells, significantly down-regulating the anti-apoptotic proteins Bcl-2 and Bcl-xL and up-regulating the pro-apoptotic protein Bax. Metformin also markedly reduced the expression of cyclin D1 and increased the numbers of apoptotic cells *in vivo*, thus inhibiting the growth of OSCC xenografts.

**Conclusions:**

Our data suggested that metformin could be a potential candidate for the development of new treatment strategies for human OSCC.

## Background

Oral squamous cell carcinoma (OSCC), which is the most common cancer of the oral cavity, is one of the leading causes of cancer-related death [1,2]. Currently, therapeutic strategies for OSCC include surgery, radiation and chemotherapy. However, despite advances in multimodal treatments, the overall survival rate of OSCC has not been improved significantly in the last several decades [2]. In addition, functional or cosmetic deficiencies and severe complications are often associated with the disease even after the treatment. Therefore, the identification of novel and effective therapeutic agents to inhibit cancer cell growth in OSCC is essential.

Metformin (1, 1-dimethylbiguanide hydrochloride) is an antihyperglycemic drug commonly used in the treatment of type 2 diabetes. Its anti-diabetic effect is mediated by the activation of AMP-activated protein kinase (AMPK), which inhibits hepatic gluconeogenesis and enhances glucose uptake in skeletal muscle [3]. In addition to its anti-diabetic properties, numerous studies have shown that metformin possesses anticancer activity, which has attracted increasing attention. In basic investigations, metformin inhibited cell proliferation in several human malignancies including gastric cancer [4], pancreatic cancer [5], medullary thyroid cancer [6], breast cancer [7] and endometrial carcinoma [8]. Metformin also suppressed tumor growth in xenograft mouse models of melanoma [9], ovarian cancer [10], prostate cancer [11] and breast cancer [12]. Furthermore, in a cancer animal model, metformin prevented tobacco carcinogen-induced lung tumorigenesis [13] and decreased the incidence and size of mammary adenocarcinomas in Her2/c-Neu transgenic mice [14]. Results from epidemiologic surveys confirm that metformin has significant effects on tumorigenesis. The use of metformin in diabetic patients was associated with significantly lower risks of cancer incidence and mortality [15]. Colorectal cancer patients with diabetes treated with metformin as part of their diabetic therapy appeared to have a superior overall survival rate [16]. However, the mechanisms underlying the suppression of cancer growth by metformin are complex, and remain relatively unknown.

Here, we demonstrated that metformin inhibited the growth of OSCC cells by blocking cell cycle progression at the G0/G1 phase and inducing apoptosis. Furthermore, metformin treatment was associated with the activation of the AMP kinase pathway and the suppression of mammalian target of rapamycin (mTOR) and S6 kinase (S6K) activation. Metformin treatment also led to a significant decrease of cyclin D1 protein level and retinoblastoma protein (pRb) phosphorylation. Cyclin-dependent kinase (CDK) 4 and CDK6 were also decreased by metformim. Moreover, a significant down-regulation of the anti-apoptotic proteins Bcl-2 and Bcl-xL and up-regulation of the pro-apoptotic protein Bax were observed in OSCC cells following metformin treatment. A colony formation assay revealed that metformin reduced the clonogenic ability of OSCC cells *in vitro*. More importantly, metformin markedly decreased the expression of cyclin D1 and increased the number of apoptotic cells in a xenograft model, showing the suppression of OSCC tumor growth *in vivo*.

## Methods

### Animals

BALB/c nude mice (male, 4 weeks of age) were purchased from Shanghai Laboratory Animal Center (Shanghai, China) and maintained in the animal care facilities of the Ninth People’s Hospital, Shanghai Jiao Tong University School of Medicine under pathogen-free conditions. Animal welfare and experimental procedures were carried out strictly in accordance with the Guide for the Care and Use of Laboratory Animals (The Ministry of Science and Technology of China, 2006) and the related ethical regulations of the hospital. All efforts were made to minimize animal suffering and to reduce the number of animals used. All experimental procedures received approval by the Laboratory Animal Care and Use Committees of the hospital.

### Cell lines and reagents

Three human OSCC cell lines (CAL27, WSU-HN6 and SCC25) were included in this study. CAL27 and SCC25 were from the American Type Culture Collection (ATCC), and WSU-HN6 was from the National Institutes of Health (NIH). All OSCC cells were provided by the Shanghai Key Laboratory of Stomatology, the Ninth’s Hospital, Shanghai Jiao Tong University School of Medicine. Metformin (1,1-dimethylbiguanide hydrochloride) was purchased from Sigma Chemical (St. Louis, MI, USA). Antibodies used for western blot analyses were from the following sources: antibodies against AMPKα, phospho-AMPKα (Thr172) (p-AMPKα), p21 and p27 were obtained from Cell Signaling Technology (Denvers, MA, USA); antibodies against Bax, Bcl-2 and Bcl-xL were obtained from BD Pharmingen (San Diego, CA, USA); anti-phospho-mTOR (Ser2448) (p-mTOR), phospho-pRb (Thr821) (p-pRb), cyclin D1, CDK4, CDK6 and phospho-S6K (p-S6K) antibodies were from Eptitomics (Burlingame, CA, USA). Anti-β-actin (clone AC-40) was purchased from Sigma. IRDye 800CW goat anti-mouse secondary antibody and goat anti-rabbit secondary antibody were obtained from LI-COR Biotechnology (Lincoln, NE, USA). PI/Rase staining buffer and the FITC Annexin V apoptosis detection kit were purchased from BD Pharmingen.

### Cell culture

CAL27 and WSU-HN6 were cultured in Dulbecco’s modified Eagle medium (DMEM) (Invitrogen, Carlsbad, CA, USA) supplemented with penicillin (100 units/ml), streptomycin (100 μg/ml) and 10% (v/v) heat-inactivated fetal bovine serum (FBS) (Invitrogen). SCC25 was cultured in F12/DMEM (Invitrogen) supplemented with the same concentrations of FBS and penicillin and streptomycin. Cells were incubated at 37°C in a humidified atmosphere containing 5% CO_2_.

### Cell proliferation assay

Human OSCC cells (5 × 10^4^ cells/well) were plated into 12-well plates. After 24 hours (h), cells were treated with metformin at the indicated concentrations or the same volume of culture medium. After incubation with metformin for 24, 48 or 72 h, cells were extensively rinsed in Dulbecco’s phosphate buffered saline (PBS) to remove any loosely attached or floating cells. The cells were then harvested by trypsinization and the cell number was determined using a hemocytometer.

### Cell clonogenic assay

Cells were seeded into 6-well plates in triplicates at a density of 1000 cells/well in 2 ml of medium containing 10% FBS. After 24 h, cultures were replaced with fresh culture medium containing the indicated concentrations of metformin in a 37°C humidified atmosphere with 95% air and 5% CO2, and grown for 3 weeks. The culture medium was changed once every 3 days. The cell clones were stained for 15 min with a solution containing 0.5% crystal violet and 25% methanol, followed by three rinses with tap water to remove excess dye. Colonies consisting of >50 cells were counted under a microscopy.

### Cell cycle and apoptosis analysis

Tumor cells (2 × 10^5^ cells/well) were seeded in 6-well plates. After 24 h, the medium was removed and replaced by fresh culture medium containing 0 mmol/L (mM) or 20 mM metformin for different time. The cell cycle was analyzed by measuring the amount of propidium iodide (PI)-labeled DNA in ethanol-fixed cells. In brief, cells were treated for 24 h, harvested by trypsinization and fixed with cold 70% ethanol. Cells were then stained for total DNA content with PI/Rase staining buffer according to the manufacturer’s instructions. Cell cycle distribution was analyzed using a flow cytometer (Becton Dickinson, San Jose, CA, USA) and ModFit software. Apoptotic and necrotic cell death were analyzed by double staining with FITC-conjugated Annexin V and PI, which is based on the binding of Annexin V to apoptotic cells with exposed phosphatidylserine and PI labeling of late apoptotic/necrotic cells with membrane damage. Tumor cells were treated for 24 and 48 h. Staining was performed according to the manufacturer’s instructions. Apoptosis was analyzed by flow cytometry, and data were processed with the FlowJo software.

### Western blot analysis

Tumor cells were seeded in a 6-well plate at a density of 5 × 10^5^ cells per well. After 24 h, the medium was replaced with fresh culture medium containing 0 mM or 20 mM metformin for different times. Cells were collected and lysed in RIPA buffer (150 mM NaCl, 10 mM Tris–HCl, pH 8.0, 1% Nonidet P-40 (NP-40), 0.5% deoxycholic acid, 0.1% SDS, 5 mM EDTA) containing 0.7% phenylmethylsulfonyl fluoride (PMSF), 0.2% aprotinin, 0.2% leupeptin, and sodium metavanadate. Samples (50 μg protein) were incubated at 100°C for 5 min, separated on 10% (w/v) SDS-PAGE gels, and electrophoretically transferred to a PVDF membrane (Bio-Rad, Hercules, CA, USA). Nonspecific sites were blocked with a solution containing 5% non-fat milk powder in TBS/Tween20 (TBS/T) for 2 h at room temperature. The membrane was probed with antibodies against β-actin, AMPKα, pAMPKα, P21, P27, Bax, Bcl-2, Bcl-xL, pmTOR, pRb, cyclin D1, CDK4, CDK6 and pS6K in TBS/T containing 5% bovine serum albumin (BSA) overnight at 4°C, and then incubated with IRDye 800CW goat anti-mouse secondary antibody or goat anti-rabbit secondary antibody at a dilution of 1:10000. Antibody-antigen complexes were detected using the Odyssey® Infrared Imaging system (LI-COR Biosciences, Lincoln, NE, USA).

### *In vivo* anti-tumor activity

For xenograft implantation, a total of 2 × 10^6^ CAL27 cells/mouse were injected subcutaneously into the back next to the right hind limb, and permitted to grow until palpable. Then mice were randomly assigned into control and treated groups and treatment was initiated. The metformin treated group received oral administration of metformin in drinking water (200 μg/ml) for 15 days, whereas the control group received drinking water only. Tumors were measured every 3 days with vernier calipers and tumor volumes were calculated according to the following formula: tumor volume (mm^3^) = *a* × *b*^2^ × 0.52, where *a* is the longest diameter and *b* is the shortest diameter. Body weight of the mice was also recorded. At the end of the experiments, tumor-bearing mice were sacrificed, and tumors were weighed after being separated from the surrounding muscles and dermis. Finally, the tumors were fixed with 4% phosphate-buffered paraformaldehyde and embedded in paraffin.

### TUNEL (terminal deoxynucleotidyl transferase (TdT)-mediated nick end labeling) staining

Paraffin-embedded tumor samples were assayed for DNA fragmentation using a TUNEL assay with the *In Situ* Cell Death Detection Kit (Roche Molecular Biochemicals, Indianapolis, IN, USA). In brief, 5-μm-thick paraffin sections of the tumor were deparaffinized in xylene and rehydrated in decreasing concentrations of ethanol. Sections were rinsed in distilled water and incubated in 3% hydrogen peroxide in methanol for 5 min to block endogenous peroxidase activity. Tissue sections were then incubated in 20 μg/ml proteinase K (DAKO Corporation, Carpinteria, CA, USA) for 15 min, washed with PBS, incubated in equilibration buffer and then in TdT enzyme solution in a humidified chamber at 37°C for 60 min. The sections were subsequently rinsed in PBS, and then incubated with streptavidin-peroxidase conjugate for 30 min. Peroxidase activity was detected by application of DAB (Vector Laboratories, Burlingame, CA, USA). Apoptotic cells were identified by a dark brown nuclear stain observed under a light microscope. A total of 10 tissue sections were analyzed for each animal.

### Immunohistochemical (IHC) staining

Cyclin D1 expression in xenograft tumor samples was determined by IHC staining. Briefly, 5-μm thick paraffin-embedded tumor sections were deparaffinized in xylene and rehydrated in decreasing concentrations of ethanol. Sections were subjected to heat-induced antigen-retrieval in citric acid buffer (pH 7.0) for 20 min, blocked in 5% normal goat serum for 30 min, and incubated in 3% hydrogen peroxide to suppress endogenous peroxidase activity. Sections were then treated with an anti-cyclin D1 (Epitomics) antibody at a dilution of 1:150 at 4°C overnight, followed by peroxidase-conjugated goat anti-rabbit antibody for 1 h at room temperature. Finally, sections were developed in a substrate solution of DAB (Vector Laboratories) and counter-stained with hematoxylin. All sections were examined under light microscopy.

### Statistical analysis

Each experiment or assay was performed at least three times, and representative examples are shown. Data were reported as means ± SD. The statistical significance of the differences was analyzed by Student’s *t*-test. The value of *p* < 0.05 was considered significant.

## Results

### Metformin inhibits the proliferation of OSCC cells and reduces colony formation *in vitro*

**Figure 1:**
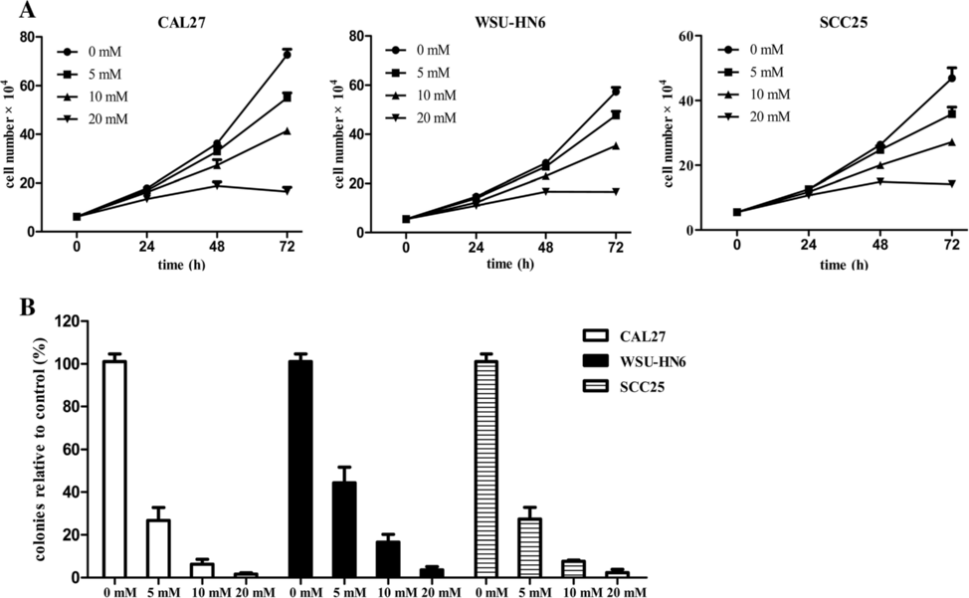
**Metformin inhibits OSCC cell proliferation and colony formation.** (**A**) 5 × 10^4^ cells/well human OSCC cells (CAL27, WSU-HN6, and SCC25) were plated onto 12-well plates and incubated at 37°C with 5% CO_2_. After 24 h, the culture medium was replaced with fresh culture medium containing 0 mM, 5 mM, 10 mM or 20 mM metformin for different time. Cell numbers were determined using a hemocytometer at each indicated time point. (**B**) Human OSCC cells (CAL27, WSU-HN6, and SCC25) were grown in 6-well plates (1000 cells/well). After 24 h, the culture medium was replaced with fresh culture medium containing 0 mM, 5 mM, 10 mM or 20 mM metformin every 3 days for 3 weeks. Cell colonies were stained and counted as described in the Methods section. Data are representative of three independent experiments.

To evaluate the growth inhibitory effect of metformin on human OSCC cells *in vitro*, three OSCC cell lines were included in our study: CAL27, WSU-HN6 and SCC25. Cells were seeded in 12-well plates and treated with or without 5, 10 and 20 mmol/L (mM) metformin for different time. Cell numbers were then determined by a hemocytometer. As shown in Figure 1A, metformin significantly inhibited proliferation in all three OSCC cell lines in a time- and dose-dependent manner. The ability of these three cell lines to form colonies on 6-well cell culture plates in the presence or absence of metformin was examined for a period of 3 weeks. Metformin significantly reduced colony formation at concentrations as low as 5 mM (Figure 1B). The inhibitory effect of metformin on colony formation was also dose-dependent, as shown in Figure 1B. At the highest concentration of 20 mM metformin, colony formation was reduced over 90% as compared to the untreated controls (0 mM). Taken together, these results indicate that metformin inhibits the growth of OSCC cells.

### Metformin induces OSCC cell cycle arrest

**Figure 2:**
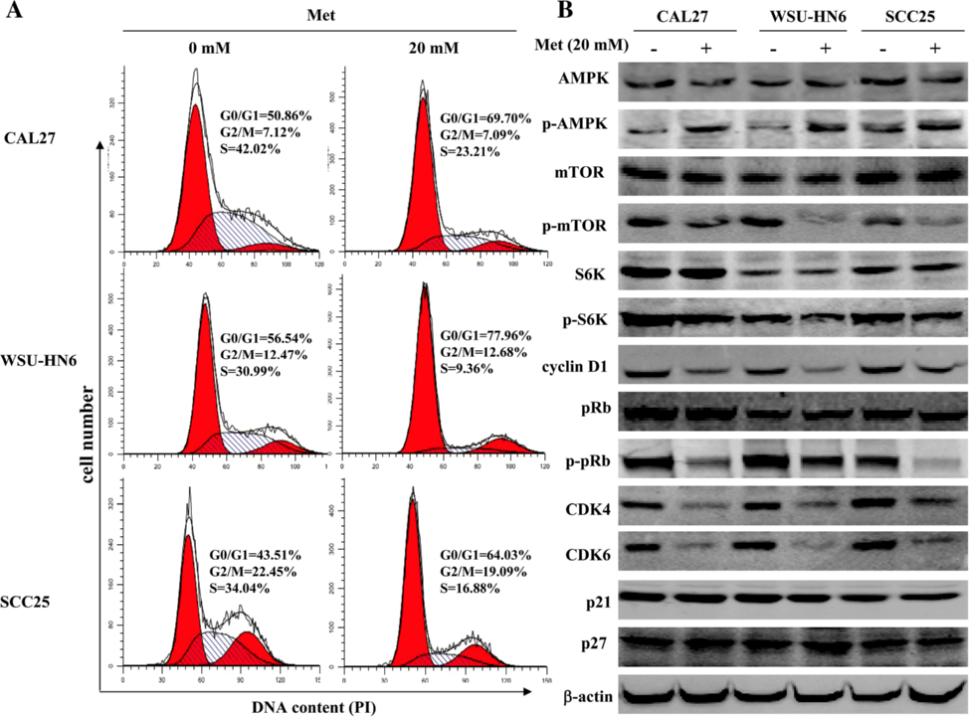
**Metformin blocks cell cycle progression at the G0/G1 phase.** Human OSCC cells (CAL27, WSU-HN6, and SCC25) were grown in 6-well plates (2 × 10^5^ cells/well). After 24 h, the culture medium was removed and replaced with fresh culture medium containing 0 mM or 20 mM metformin for an additional 24 h. (**A**) Cell cycle progression in OSCC cells was assessed by flow cytometry. (**B**) The expression of related cell-cycle regulatory proteins in arrested and proliferating OSCC cells treated with or without metformin was assessed by immunoblotting. One representative experiment out of three is shown.

The possible effect of metformin on cell cycle progression in OSCC cells was examined by flow cytometry. Treatment of proliferating CAL27, WSU-HN6 and SCC25 cells with 20 mM metformin for 24 h caused delayed entry into S phase and induced G0/G1 arrest. Metformin treatment increased the proportion of cells in the G0/G1 phase in all three OSCC cell lines compared to control cells (69.7% vs. 50.86% in CAL27, 77.96% vs. 56.54% in WSU-HN6, and 64.03% vs. 43.51% in SCC25) (Figure 2A). The proportion of OSCC cells in the S phase decreased accordingly, whereas there was no significant change in the number of cells in the G2/M phase.

The expression of various cell-cycle-related molecules in OSCC cells treated with or without 20 mM metformin for 24 h was then examined by western blot. The most remarkable change was the loss of cyclin D1, a key protein implicated in the transition from the G0/G1 to the S phase (Figure 2B). Increased levels of p-AMPKα in metformin-treated cells indicated the activation of the AMPK pathway. The protein levels of p-mTOR, p-S6K and p-pRb also decreased dramatically in response to metformin treatment (Figure 2B). Analysis of the expression of other cell-cycle-related proteins involved in the G0/G1 transition revealed an obvious decrease of CDK4 and CDK6 levels in OSCC cells treated with metformin. However, no significant changes were detected in the expressions of p21 and p27. These results clearly demonstrate that metformin affects the expression and the phosphorylation of key cell cycle regulatory proteins leading to G0/G1 arrest in human OSCC cells.

### Metformin induces apoptosis of OSCC cells

**Figure 3:**
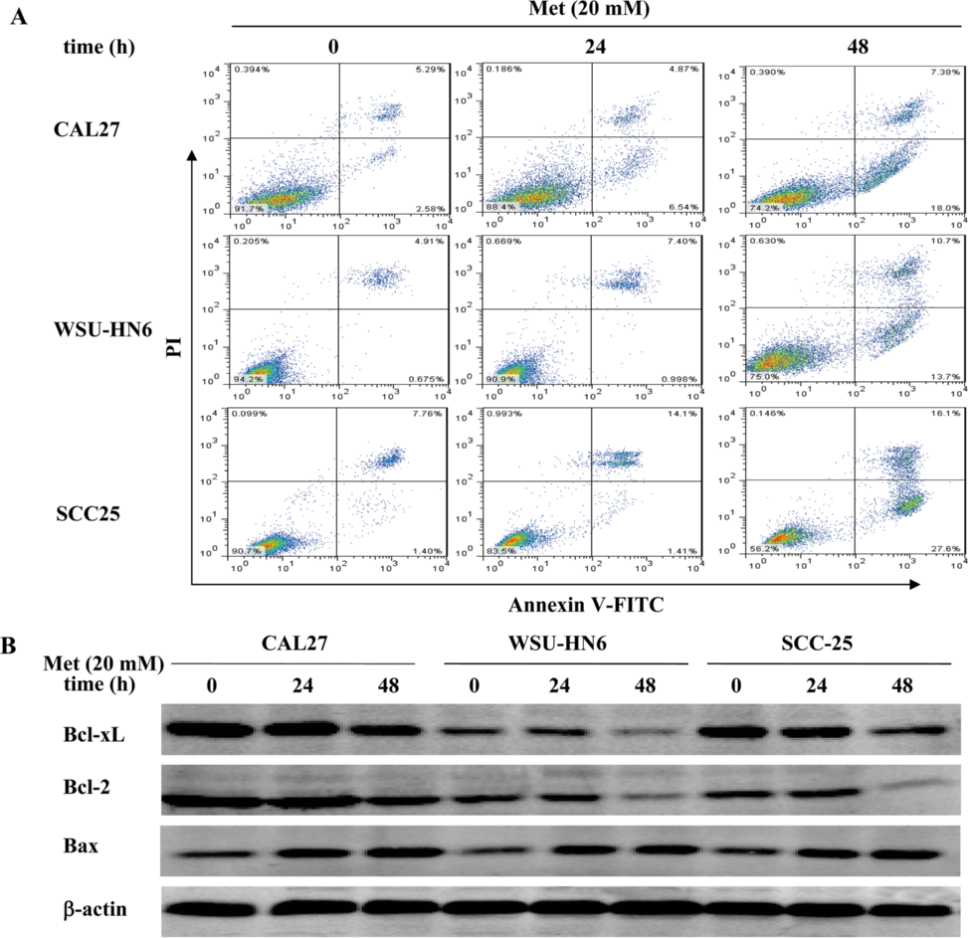
**Metformin induces apoptosis of OSCC cells.** Human OSCC cells (CAL27, WSU-HN6, and SCC25) were grown in 6-well plates (2 × 10^5^ cells/well). After 24 h, the culture medium was removed and replaced with fresh culture medium containing 0 mM or 20 mM metformin for another 24 h or 48 h. (**A**) Apoptosis of OSCC cells was analyzed by flow cytometry. (**B**) The expression of the anti-apoptotic proteins Bcl-2 and Bcl-xL and the pro-apoptotic protein Bax in OSCC cells treated with or without metformin was assessed by western blot. Data is representative of three independent experiments.

To determine whether metformin induced apoptosis, OSCC cells were treated with or without 20 mM metformin for 24 h and 48 h and analyzed by flow cytometry. The results showed that metformin induced a dramatic increase in the proportion of apoptotic tumor cells 48 h after treatment in CAL27, WSU-HN6 and SCC25 cells (25.4%, 24.4% and 43.7%, respectively), with 11.4%, 8.4% and 15.5% of apoptotic cells at 24 h after treatment, respectively (Figure 3A). The percentages of apoptotic tumor cells in the control groups of CAL27, WSU-HN6 and SCC25 were 7.8%, 5.5% and 9.2%, respectively (Figure 3A). To investigate the mechanisms underlying the apoptosis-inducing effect of metformin in OSCC cells, the levels of apoptosis-related proteins such as Bcl-2, Bcl-xL and Bax were measured in total protein from tumor cells treated with or without 20 mM metformin for 24 h and 48 h by western blot. Metformin significantly down-regulated the expression of the anti-apoptotic proteins Bcl-2 and Bcl-xL and up-regulated the pro-apoptotic protein Bax (Figure 3B). These results indicate that an apoptotic mechanism is implicated in the metformin-induced inhibition of proliferation in OSCC cells.

### Metformin impairs OSCC growth *in vivo*

**Figure 4:**
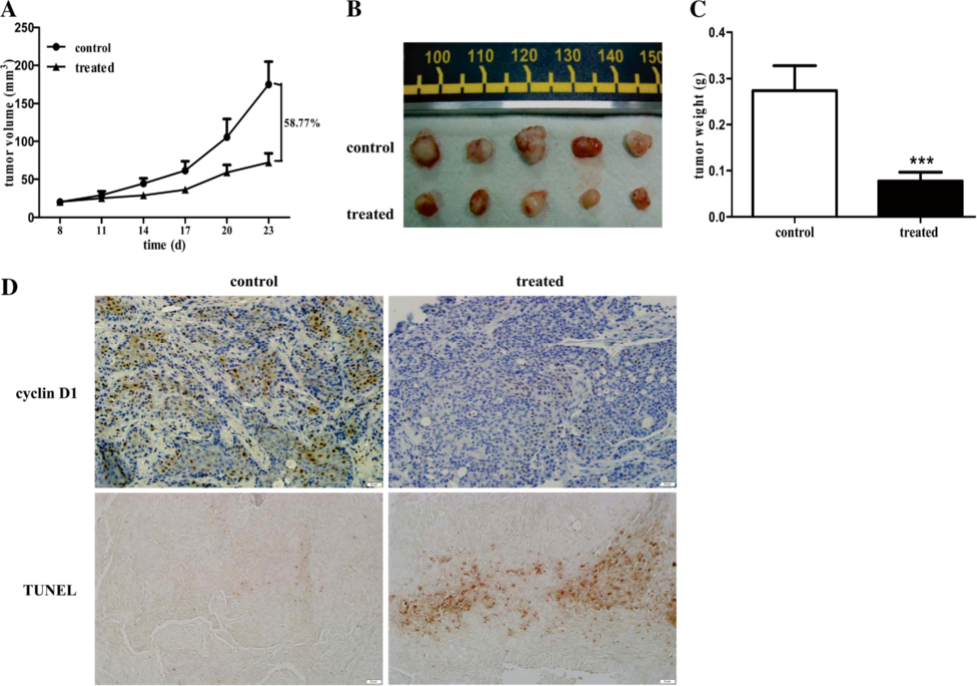
***In vivo *anti-tumor effects of metformin in OSCC xenografts in nude mice.** A total of 2 × 10^6^ CAL27 cells/mouse were injected subcutaneously into the back next to the right hind limb, and permitted to grow until palpable. Metformin was administered orally for 15 days; control mice received drinking water only. (**A**) Graphs represent the average tumor volumes of CAL27 xenografts in mice from the control and metformin–treated groups. (**B**) Representative images of tumors from mice in the two groups. (**C**) Weight of tumors from the control and metformin-treated groups. (**D**) Cyclin D1 expression and apoptotic tumor cells in tumors from mice treated with or without metformin were assessed by IHC and TUNEL staining, respectively (original magnification is 200×). Five mice were included for each group, and results are representative of three experiments. (***p < 0.001).

Finally, we investigated whether metformin could prevent OSCC progression *in vivo*. The CAL27 cell line was randomly selected for the establishment of the OSCC xenograft nude mouse model. After solid tumors were palpable (day 8), mice were randomly assigned into control and treated groups. Metformin was administered orally to the treated group in drinking water (200 μg/ml), whereas the control mice only received fresh drinking water. During our experiments, no obvious side effects were observed in mice treated with metformin (data not shown). Tumor volumes and tumor weights were measured. Consistent with our *in vitro* results, oral administration of metformin led to a substantial inhibition of tumor growth by 58.77% (Figure 4A). CAL27 xenograft nude mice treated with metformin had a significantly reduced tumor burden compared with control mice, as reflected in the obvious reduction in the sizes and weights of tumors from metformin-treated mice (Figure 4B and 4C). The mean weights of the excised tumors were approximately 69.3% lower in mice treated with metformin than in untreated mice. To determine whether metformin affected cyclin D1 protein levels and apoptosis of tumor cells *in vivo*, we further analyzed cyclin D1 expression and apoptotic tumor cells in xenograft tumors by IHC and TUNEL staining, respectively. Metformin markedly reduced the expression of cyclin D1 and increased the number of apoptotic tumor cells compared to the untreated controls (Figure 4D). Thus, similar to the *in vitro* results, metformin impairs the growth of OSCC cells *in vivo* through the induction of cell cycle arrest and apoptosis.

## Discussion

As a stable, inexpensive and highly effective oral drug, metformin has been used for the treatment of type 2 diabetes for several decades. It stimulates glucose uptake and increases fatty acid oxidation in muscle and liver with no adverse effects [3,17]. Recent data indicate that metformin can protect from cancer and inhibit the proliferation of several types of cancer cells *in vitro* and *in vivo*, such as breast cancer [18], gastric cancer [4], pancreatic cancer [19], and thyroid cancer [6]. The anti-tumor effects of metformin have been investigated in different types of adenocarcinoma; however, its effects on squamous cell carcinoma, a malignant tumor of epidermal keratinocytes that invades the dermis, have not yet been well defined. Adenocarcinoma and squamous cell carcinoma can differ significantly in their symptoms, natural history, prognosis, and response to treatment owing to differences in cellular origin. In the present study we focused on the effects of metformin on OSCC, a common squamous cell carcinoma of the head and neck. The present findings are significant because 1) we demonstrate for the first time that metformin exerts potent anti-OSCC effects both *in vitro* and *in vivo*; 2) metformin induces cell cycle arrest at the G0/G1 phase and apoptosis of OSCC cells associated with the modulation of cell cycle-regulatory and apoptosis-related protein expression. CDK inhibitors such as p21 and p27 have been shown to play an important role in the inhibitory effects of metformin in previous studies [18,20]. However, in the present study, we did not observe significant changes of these proteins in OSCC cells following metformin treatment. This discrepancy could be due to the differences in the properties of the different types of cancer cells.

A previous study showed that specific cyclin/CDK complexes are activated at different intervals during the cell cycle and complexes of CDK4 and CDK6 with cyclin D1 are required for G1 phase progression [21]. Down-regulation of cyclin D1 in response to metformin has been shown in several cancer cell lines including breast cancer [18] and prostate cancer [11] cells. The effects of metformin on the catalytic subunits of cyclin D1, CDK4 and CDK6 in OSCC cells, however, remain unknown. In the present study, metformin blocked cell cycle progression at the G0/G1 phase, which was correlated with a remarkable decrease in the expression of cyclin D1 and phosphorylation of pRb, two major cell-cycle regulators. Cyclin D1 binds to and activates CD4/CDK6, which then phosphorylates pRb. Upon phosphorylation, pRb releases the transcription factor E2F, which activates the transcription of genes required for G1/S phase transition [22]. Cyclin D1 gene amplification and overexpression are observed in several types of human cancer including OSCC [23-25]. Furthermore, overexpressed cyclin D1 is associated with enhanced tumor growth and chemotherapy resistance [24,26]. Thus, cyclin D1 is a potential molecular target for the treatment of OSCC. In addition to its effect on cyclin D1, metformin strongly inhibits the phosphorylation of pRb in OSCC cells, blocking the activation of E2F. Activation of E2F by disruption of the Rb tumor suppressor pathway is a key event in the development of many human cancers. Increased expression of E2F is associated with malignant transformation in OSCC, and down-regulation of this transcription factor is associated with induction of apoptosis and cell cycle arrest in OSCC cells [27,28]. Therefore, our results suggest that metformin could be developed as a potential therapeutic agent to block the progression of OSCC.

In the present study, metformin activated the AMPK pathway and inhibited S6K and mTOR phosphorylation in OSCC cells, suggesting that the mTOR pathway may be involved in mediating the effect of metformin in these cells. However, the role of AMPK in the activation of mTOR signaling is the subject of controversy. Using siRNA against the two catalytic subunits of AMPK, Ben Sahra et al. demonstrated that the anti-proliferative effect of metformin was mediated by the mTOR pathway independently of AMPK [11]. On the other hand, Zakikhani et al. showed that metformin inhibited cell growth via the α1 AMPK subunit in MCF-7 breast cancer cells [29]. Although our results clearly showed the growth inhibitory effect of metformin in OSCC, the involvement of the AMPK pathway in the anti-tumor effect of metformin on OSCC remains to be elucidated. Moreover, because metformin is known to play a role in the control of cell metabolism, it would be interesting to determine whether the metabolic consequences of metformin are related to its anti-proliferative effects.

In addition to the effect of metformin on the cell cycle, we examined whether the anti-neoplastic effect of this agent is mediated by the induction of apoptosis. Our flow cytometry results demonstrated that metformin significantly induced apoptosis in all three OSCC cells lines. These findings were further confirmed by our western blot results showing a significant down-regulation of the anti-apoptotic proteins Bcl-2 and Bcl-xL and the up-regulation of the pro-apoptotic protein Bax. Several death and survival genes, such as Bcl-2 or Bax, which are regulated by extracellular factors, are involved in apoptosis [30]. When the ratio of pro-apoptotic Bcl-2 family members (Bax, Bad) to anti-apoptotic Bcl-2 family members (Bcl-2, Bcl-xL and Mcl-1) increases, pores form in the outer mitochondrial membrane, liberating apoptogenic mitochondrial proteins to activate caspases and induce apoptosis [31]. Data concerning the effect of metformin on apoptosis in cancer cells are limited and controversial. A recent study indicated that metformin suppressed the growth of human head and neck squamous cell carcinoma mainly via G1 arrest, which coincided with a decrease in the protein levels of CDKs, cyclins and CDK inhibitors [32]. Ben Sahra et al. also showed that metformin blocked the cell cycle in the G0/G1 phase in prostate cancer cells and did not induce apoptosis [11]. In contrast, metformin has been shown to promote apoptosis in pancreatic cancer [19] and melanoma [9] cells. This discrepancy between studies regarding the effect of metformin on apoptosis may be the result of variations in experimental conditions, cell-specific functions and/or different cell origin, and suggests that further investigation is necessary. Moreover, Hirsch et al. [33] reported that low doses of metformin could inhibit cellular transformation and selectively kill cancer stem cells in four genetically different types of breast cancer, thus inhibited the tumor growth both *in vitro* and *in vivo*. Whether similar mechanisms also contribute to the anti-cancer effect of metformin in OSCC still needs to be identified in our further study.

Although the doses of 20 mM metformin used in our *in vitro* study are similar to those used in prior studies on gastric cancer [4], melanoma [9] and breast cancer [29], one can still argue that these doses are above physiological levels. Indeed, the concentration of metformin in the blood of type 2 diabetic patients treated with the drug is approximately 30 ~ 60 μmol/L [34], which indicates that the doses used in our study exceeded the therapeutic level by 300-fold. However, it has been reported that metformin accumulates in tissues at concentrations similar to the dose used in our experiments [35,36]. Moreover, tumor cells in culture are grown under high concentrations of glucose and 10% FBS, which results in excessive growth stimulation. This may also contribute to the high dose of metformin required to exert anti-tumor effects in a cell culture system compared to the dose used in patients with diabetes. Furthermore, according to the study of Ben Sahra et al., the doses of 1 to 3 mg/day metformin caused no side effect in mice, which was equal to the dosage used for patients [11], we obtained a strong inhibition of OSCC tumor growth *in vivo*. 200 μg/ml metformin administered orally significantly decreased OSCC growth in a xenograft model. This result is of particular importance as it is the first time that metformin is shown to inhibit OSCC tumor growth *in vivo*.

## Conclusions

The present study used a cell culture system and a tumor xenograft mouse model to demonstrate for the first time that metformin effectively inhibits OSCC cell proliferation and tumor growth *in vitro* and *in vivo*. Our results suggest that metformin could be a potential candidate for the development of novel treatment strategies for human OSCC, which warrants further investigation.

## Abbreviations

OSCC: Oral squamous cell carcinoma; Metformin: 1,1-dimethybiguanide hydrochloride; AMPK: AMP-activated protein kinase; mTOR: Mammalian target of rapamycin; S6K: S6 kinase; pRb: Retinoblastoma protein; CDK: Cyclin-dependent kinase; TUNEL: Terminal deoxynucleotidyl transferase (TdT)-mediated nick end labeling) staining; PI: Propidium iodide; IHC: Immunohistochemical.

## Competing interests

The authors declare that they have no competing interests.

## Authors' contributions

FXC and QQL designed and coordinated the study. QQL and DH carried out all the experiments, performed the statistical analysis and drafted the manuscript. SQH and ZJS helped with the animal experiments. MY contributed to the cell culture and the IHC staining. All authors read and approved the final manuscript.

## Pre-publication history

The pre-publication history for this paper can be accessed here:

http://www.biomedcentral.com/1471-2407/12/517/prepub
